# The long-term effects of consecutive COVID-19 waves on mental health

**DOI:** 10.1192/bjo.2023.620

**Published:** 2023-12-19

**Authors:** Jan Sebastian Novotný, Juan Pablo Gonzalez-Rivas, Šárka Kunzová, Mária Skladaná, Anna Pospíšilová, Anna Polcrová, Maria Vassilaki, Jose Ramon Medina-Inojosa, Francisco Lopez-Jimenez, Yonas Endale Geda, Gorazd Bernard Stokin

**Affiliations:** Institute for Molecular and Translational Medicine, Faculty of Medicine and Dentistry, Palacký University Olomouc, Czech Republic; International Clinical Research Centre, St. Anne's University Hospital Brno, Czech Republic; and Department of Global Health and Population, Harvard T.H. Chan School of Public Health, Harvard University, USA; International Clinical Research Centre, St. Anne's University Hospital Brno, Czech Republic; International Clinical Research Centre, St. Anne's University Hospital Brno, Czech Republic; and Second Department of Internal Medicine, St. Anne's University Hospital Brno and Masaryk University, Brno, Czech Republic; International Clinical Research Centre, St. Anne's University Hospital Brno, Czech Republic; and Research Centre for Toxic Compounds in the Environment (RECETOX), Masaryk University, Brno, Czech Republic; Division of Epidemiology, Department of Quantitative Health Sciences, Mayo Clinic, Rochester, Minnesota, USA; Division of Preventive Cardiology, Department of Cardiovascular Medicine, Mayo Clinic, Rochester, Minnesota, USA; and Marriot Heart Disease Research Program, Mayo Clinic, Rochester, Minnesota, USA; Division of Preventive Cardiology, Department of Cardiovascular Medicine, Mayo Clinic, Rochester, Minnesota, USA; Department of Neurology, Barrow Neurological Institute, Phoenix, Arizona, USA; and Franke Global Neuroscience Education Center, Barrow Neurological Institute, Phoenix, Arizona, USA; Institute for Molecular and Translational Medicine, Faculty of Medicine and Dentistry, Palacký University Olomouc, Czech Republic; and Department of Neurology, Gloucestershire Hospitals NHS Foundation Trust, Gloucester, UK

**Keywords:** COVID-19, longitudinal, stress levels, depressive symptoms, stressors

## Abstract

**Background:**

Although several studies have documented the impact of the COVID-19 pandemic on mental health, the long-term effects remain unclear.

**Aims:**

To examine longitudinal changes in mental health before and during the consecutive COVID-19 waves in a well-established probability sample.

**Method:**

An online survey was completed by the participants of the COVID-19 add-on study at four time points: pre-COVID-19 period (2014–2015, *n* = 1823), first COVID-19 wave (April to May 2020, *n* = 788), second COVID-19 wave (August to October 2020, *n* = 532) and third COVID-19 wave (March to April 2021, *n* = 383). Data were collected via a set of validated instruments, and analysed with latent growth models.

**Results:**

During the pandemic, we observed a significant increase in stress levels (standardised *β* = 0.473, *P* < 0.001) and depressive symptoms (standardised *β* = 1.284, *P* < 0.001). The rate of increase in depressive symptoms (std. covariance = 0.784, *P* = 0.014), but not in stress levels (std. covariance = 0.057, *P* = 0.743), was associated with the pre-pandemic mental health status of the participants. Further analysis showed that secondary stressors played a predominant role in the increase in mental health difficulties. The main secondary stressors were loneliness, negative emotionality associated with the perception of COVID-19 disease, lack of resilience, female gender and younger age.

**Conclusions:**

The surge in stress levels and depressive symptoms persisted across all three consecutive COVID-19 waves. This persistence is attributable to the effects of secondary stressors, and particularly to the status of mental health before the COVID-19 pandemic. Our findings reveal mechanisms underlying the surge in mental health difficulties during the COVID-19 waves, with direct implications for strategies promoting mental health during pandemics.

Several studies have reported changes in mental health immediately following the onset of the COVID-19 pandemic.^1,2^ To date, however, only a limited number of studies have addressed the long-term effects of consecutive COVID-19 waves on mental health.^3–7^ Some of these studies suggest that COVID-19 acts as a chronic stressor directly by the SARS-CoV-2 infection, as well as indirectly by changing the socioeconomic, lifestyle and other circumstances of those experiencing the COVID-19 pandemic for longer than a year.^6^

Recent studies of the long-term effects of the COVID-19 pandemic on mental health reported significantly higher levels of stress,^8^ anxiety^3,4^ and depressive symptoms^3,4,7^ during the pandemic compared with the pre-COVID-19 period. Furthermore, younger age,^6,9^ female gender,^3,10^ history of prior psychological distress,^11^ worries and negative emotions about COVID-19,^9,12^ and feelings of loneliness^6,10^ were identified as putative risk factors for developing mental health concerns during the COVID-19 pandemic. Some studies also suggested that adverse mental health outcomes reported during the COVID-19 pandemic might be long-lasting because they have been noted in people more than a year following SARS-CoV-2 infection.^13^ Collectively, these observations are therefore suggestive of consecutive COVID-19 waves contributing to long-term adverse mental health outcomes. The long-term trajectories and causes of mental health difficulties, however, remain unclear. Based on previous findings, we examine a probability population-based sample to test the hypothesis that changes in mental health during the consecutive COVID-19 waves are persistent, and are caused by secondary stressors acting before as well as during the COVID-19 pandemic.

## Method

### Study design and study population

This is a single-centre, longitudinal COVID-19 ancillary study performed with the well-established Kardiovize population-based sample representing 1% of the inhabitants of the city of Brno, Czech Republic.^12,14^ Participants of the Kardiovize study were selected randomly from databases of health insurance companies, to represent a probabilistic sample of the general population. Detailed descriptions of the recruitment processes for the Kardiovize study has been published elsewhere.^14^ The entire Kardiovize study cohort was invited by email to participate in the COVID-19 add-on study.^12^ The inclusion criterion for the analysis of the COVID-19 add-on study results was the availability of data on stress and depressive symptoms at baseline pre-COVID-19 and at least one of the COVID-19 waves. This inclusion criterion was used to ensure that all of the analysed data were longitudinal, while avoiding intercept and growth slope bias caused by a larger pre-COVID-19 sample. Although all citizens and permanent residents in the Czech Republic are registered with health insurance companies, the selection bias related to the most disadvantaged of society (for example, not responding to the invitation, not having or regularly using an email address, etc.) cannot be excluded, but has been estimated to be minimal. The COVID-19 add-on study followed the Strengthening the Reporting of Observational Studies in Epidemiology (STROBE) reporting guidelines.^15^

### Procedure

**Fig. 1:**
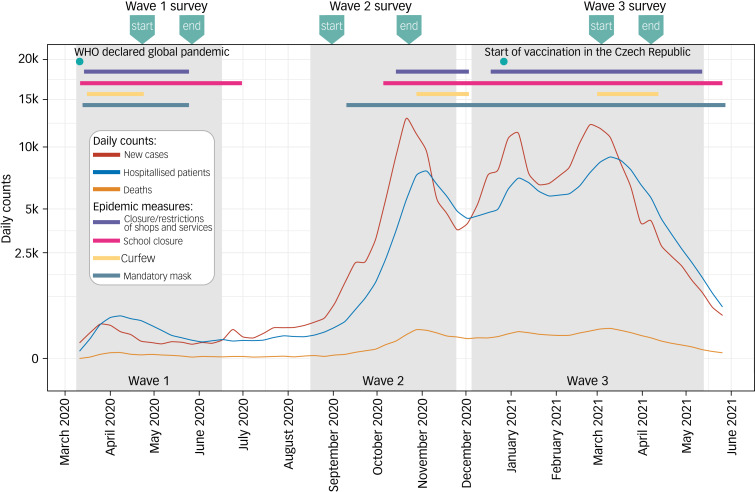
Timeline of the COVID-19 pandemic and restrictive measures in the Czech Republic. The lines show trends in daily numbers of new SARS-CoV-19 cases, SARS-CoV-19-related hospital admission and deaths over the examined time period of the pandemic (data are projected on a square-root-transformed scale). The upper horizontal bars show the time periods when key epidemiological measures were imposed. The grey sections indicate individual waves of the COVID-19 pandemic. The indicators above the plot show the time periods (start and end) of data collection in each COVID-19 wave. Source of the data: Ministry of Health of the Czech Republic (https://onemocneni-aktualne.mzcr.cz/covid-19). WHO, World Health Organization.

In the COVID-19 add-on study, we examined mental health in the same population-based sample before the COVID-19 pandemic and during the three consecutive COVID-19 waves (Fig. 1). Mental health data were collected (a) before the COVID-19 pandemic, in 2014 and 2015; (b) during the first COVID-19 wave, between 23 April and 27 May 2020; (c) during the second COVID-19 wave, between 31 August and 23 October 2020; and (d) during the third COVID-19 wave, between 4 March and 7 April 2021. All participants completed the Kardiovize COVID-19 e-questionnaire (in Supplementary Appendix 1 available at https://doi.org/10.1192/bjo.2023.620), which includes a battery of psychological scales, through an online survey administered with validated RedCap software (version 10.3.8, REDCap Consortium, Vanderbilt University, TN, USA; see https://www.project-redcap.org/).

The primary outcomes were the stress levels, measured with the Perceived Stress Scale (PSS),^16^ and the severity of depressive symptoms, measured with the Patient Health Questionnaire (PHQ).^17^ The PSS is a ten-item scale scored with a five-point Likert scale (0 ‘never’ to 5 ‘very often’), with a possible range of 0–40. Stress levels were categorised as low (score of 0–13), medium (score of 14–26) or high (score of 27–40). The reliability of the Czech version of the PSS in a single-factor approach was *ɑ* = 0.91.^18^ The cross-sectional McDonald's omega was *ω* = 0.90, 0.90 and 0.91 in the first, second and third waves of the study, respectively, and the longitudinal stability (intraclass correlation coefficient (ICC)) was 0.88. The presence and severity of depressive symptoms were assessed with the two identical items from the PHQ-9 (before the COVID-19 pandemic) and PHQ-4 (during the COVID-19 pandemic). Responses were measured with a four-point Likert scale (0 ‘not at all’ to 3 ‘almost every day’), with a possible range of 0–6 points. Despite its brevity, the PHQ-4 has proven to be a valid tool for rapid assessment of the general presence of depressive symptoms.^17,19–21^ Reliability in the original study was *ɑ* = 0.78; the cross-sectional Spearman–Brown composite reliability was r* = 0.82, 0.83 and 0.82 in the first, second and third waves of the study, respectively; and the longitudinal stability (ICC) was 0.74. Depressive symptoms were considered present if the sum of the score of the two PHQ items was ≥3. Since stress, depression, anxiety and other related terms represent different negatively connoted aspects of mental health and the general terminology is not fully established, we used the term mental health difficulties to collectively refer to moderate-to-high stress and increased severity of depressive symptoms, as an expression of the negative effects of these terms on mental health.^22–24^

To identify factors affecting mental health, we collected information on several primary and secondary stressors, using well-established measurement tools. Primary stressors are factors that are inherent and arise directly from a negative event (such as fear of contracting COVID-19 and its possible health consequences such as death, etc.). In contrast, secondary stressors are non-inherent to the event. They arise partially from the consequences of this negative event (such as lockdowns, separation from loved ones and disruption of finances) and partially from longer-term socioeconomic disadvantage and lack of resilience to adversities, etc.^25–27^ Potential risk factors were selected by the Kardiovize researchers based on literature review. A brief review of the literature supporting the inclusion of each selected factor is presented in the Supplementary Table 1.

Primary stressors included cognitive and emotional perception of COVID-19, assessed with the Brief Illness Perception Questionnaire (B-IPQ).^28^ This eight-item scale uses a ten-point Likert scale (1–10 with higher scores indicating a more threatening perception of the illness), with a possible range of 10–70, and measures cognitive and emotional perception of the COVID-19 disease as threatening across different domains. The cross-sectional reliability in the first, second and third waves of the study, respectively, was *ω* = 0.51, 0.57 and 0.54 for cognitive perception and *ω* = 0.60, 0.58 and 0.61 for emotional perception of COVID-19 disease. These values are acceptable given that the B-IPQ measures different aspects of COVID-19 perception at the item level, which increases the heterogeneity of higher-order factors.^29,30^ The longitudinal stability (ICC) was 0.71 for cognitive perception and 0.87 for emotional perception of COVID-19 disease. In agreement with the meta-analytic study about the B-IPQ,^31^ we consider the validity and reliability of the instrument to be sufficiently established. A higher B-IPQ score implies that COVID-19 illness is perceived as more threatening.

Secondary stressors included feelings of loneliness, lack of resilience and the effects of the COVID-19 pandemic and associated measures on finances and work, social contacts, sleep and physical activity. The feeling of loneliness was assessed with the UCLA 3-Item Loneliness Scale.^32^ Responses are scored on a three-point Likert scale (1 ‘seldom’ to 3 ‘often’), providing a total score from 3 to 9 points. The UCLA 3-Item Loneliness Scale showed a reliability of *ɑ* = 0.89–0.94 in the original study^33^ and *ω* = 0.75, 0.79 and 0.77 in the first, second and third waves of this study, respectively, with a longitudinal stability (ICC) of 0.81. Higher scores indicate greater feelings of loneliness. Presence of the feeling of loneliness was defined as score ≥6.

Resilience was assessed with the Connor–Davidson Resilience Scale.^34^ This short two-item instrument with a five-point Likert scale (0 ‘not at all’ to 4 ‘almost always’) and a score range of 0–8, demonstrated very good validity and reliability, with *ɑ* = 0.79.^34^ The reliability was r* = 0.70, 0.72 and 0.70 in the first, second and third waves of the study, respectively, and the longitudinal stability (ICC) was 0.84. Presence of resilience was defined as low (score of 0–5), medium (score of 6–7) or high (score of 8).

The impact of the COVID-19 pandemic on finances, work, social contacts, sleep and physical activity was measured with the Kardiovize COVID-19 e-questionnaire (Supplementary Appendix 1). The items in this section of the e-questionnaire were designed by the Kardiovize researchers based on a literature review. This section has not been validated. All participants also provided general demographic and health data.

### Statistical analysis

Missing values correspond only to respondents who dropped out in each wave. No imputation of missing values was applied. The nature of the missing data was examined with a correlation analysis of the relationship between the presence of missing values (using a binary matrix representing missingness) and the observed values. The results showed that the data are missing completely at random, as there was no relationship between missingness and the observed values (mean absolute correlation *r* = 0.046, maximum absolute correlation *r* = 0.2; all *P* > 0.05). No sensitivity analysis was performed.

Cross-sectional reliability of the instruments used in each wave of the study was verified with McDonald's omega (*ω*) and the Spearman-Brown composite reliability (r*) for the two-item scales. Long-term stability was verified with the ICC. The Wilcoxon rank-sum test and the chi-squared test were used to verify representativeness of the COVID-19 study population. Differences in the prevalence of mental health difficulties were verified with the *G*-test, with Benjamini–Hochberg correction for multiple comparisons.

Longitudinal changes in the stress levels, severity of depressive symptoms and the effects of different secondary stressors were analysed with latent growth curve models (LGCMs). LGCM simulates change as a function of time, which is represented by the latent variables, referred to as growth factors.^35–37^ A latent intercept and a latent slope (i.e. the growth factors) are estimated based on the individual trajectories. Growth factors provide an estimate of the average trajectory and the individual variation around that trajectory over time. These parameters provide an insight into the average change and the individual difference surrounding that change. The inclusion of additional time-variant and -invariant covariates then allow us to assess the effects of these stressors on the primary variable and the possible changes in its growth curve. Compared with analysis-of-variance methods, the LGCM also allows for the inclusion of respondents with missing values at some time points in the study (if there is more observed information than estimated information, i.e. a sufficient number of cases have at least three repeated measures), thereby increasing the overall *N* and the power of the results.^38^

As part of the analysis, we first modelled four types of growth (linear, quadratic, cubic and relative change) for the two primary outcomes, and compared their overall fit. Linear growth was specified by constraining the loadings of the latent growth parameters on the observed outcomes to assume incremental change per increase in unit time. Quadratic and cubic growths were modelled by fixing factor loadings of the quadratic and cubic terms to assume exponential change. Growth based on relative change was modelled by fixing the first loading to 0 and the last loading to 1, with the other loadings being freely estimated by the growth function itself. By convention, the intercept and slope parameters were allowed to co-vary.^39–41^ Model fits were evaluated primarily based on the Akaike information criterion/Bayesian information criterion, with further checking of Comparative Fit Index, Tucker–Lewis index and root-mean-square error of approximation values. We then analysed the selected growth model in the context of the primary outcomes, stress levels and severity of depressive symptoms. Finally, we tested the effect of secondary stressors on the observed growth curve of both primary outcomes by adding the stressors as covariates to the LGCM. To keep the model parsimonious, only stressors with significant influence were retained in the final model. We also verified that the fit of the final model is better than the base full model with all predictors included (Supplementary Table 2). Given the non-normal distribution of some stressor scores, we have used and report a robust estimator (maximum likelihood estimation with Huber–White s.e.). All two-sided *P* < 0.05 were considered to be significant. Data analysis was performed with RStudio (version 2022.02.3, R version 4.2.0 for Windows, Posit, PBC, Boston, MA, USA; see https://posit.co/download/rstudio-desktop/).

### Ethics statement

The authors assert that all procedures contributing to this work comply with the ethical standards of the relevant national and institutional committees on human experimentation and with the Helsinki Declaration of 1975, as revised in 2008. All procedures involving human patients were approved by the Internal Review Board and the St. Anne's University Hospital ethics committee (reference number 28 V/2019, permission granted 28 April 2020). All participants provided written informed consent to participate in this study.

## Results

### Sample characteristics and representativeness

A total of 1823 Kardiovize study participants who completed the pre-COVID-19 baseline assessment were electronically invited to join the COVID-19 add-on study; 43% (*N* = 788) of the participants were ultimately enrolled into the COVID-19 add-on study and completed the survey during the first COVID-19 wave. The COVID-19 add-on study sample in general maintained the representativeness of the original Kardiovize study population. Specifically, gender (*P* = 0.98), education (*P* = 0.99), job position (*P* = 0.99), pre-pandemic stress levels (*P* = 0.50) and severity of depressive symptoms (*P* = 0.44) were all comparable between the Kardiovize and the COVID-19 add-on study cohorts. Only the age differed between the two cohorts, with a difference in mean age of 1.32 years (mean: Kardiovize cohort, 47.3 (95% CI 46.83–47.78) years; COVID-19 add-on study cohort, 45.98 (95% CI 45.17–46.78) years; *P* = 0.004).

Compared with the first COVID-19 wave, the online survey was completed by 532 (67.5%, including 14 new participants) and 383 (48.6%) of the COVID-19 add-on study participants during the second and third COVID-19 wave, respectively.

Given the inclusion criterion of the available longitudinal data from pre-COVID-19 pandemic and at least one COVID-19 wave, the final sample included in the LGCM analyses consisted of 802 participants. Of these, 270 (33.7%) participants engaged in the first COVID-19 wave only, 143 (17.8%) in the first and second waves, six (0.7%) in the second wave only, eight (1%) in the second and third waves, and 375 (46.8%) in all three COVID-19 waves. This yields 526 (65.6%) participants with at least three repeated measures, allowing satisfactory identification of linear growth trajectories. The basic demographic characteristics within each wave are shown in Table 1.

**Table 1:** Demographics of the COVID-19 add-on study population-based sample

	Kardiovize sample	Wave 1	Wave 2	Wave 3
*N*	1823	788	532	383
Dropped out^a^		0	270	149
Returned^a^		0	14	0
Age^b^	54.7 (54.2–55.2)	52.9 (52.1–53.8)	52.9 (51.9–54.0)	53.2 (52.0–54.4)
Gender^c^				
Male	805 (44.1%)	359 (45.6%)	235 (44.2%)	165 (43.1%)
Female	1018 (55.9%)	429 (54.4%)	297 (55.8%)	218 (56.9%)
Education^c^^,^^d^				
Without GCSE	362 (19.8%)	106 (13.5%)	61 (11.5%)	42 (11%)
With GCSE	704 (38.7%)	334 (42.4%)	220 (41.3%)	158 (41.2%)
University	757 (41.5%)	348 (44.1%)	251 (47.2%)	183 (47.8%)

GCSE, General Certificate of Secondary Education.

a. Compared with previous wave.

b. Presented as mean (95% CI).

c. Presented as *n* (%).

d. University education includes higher vocational school, Bachelor, Master and Doctoral degrees.

### Choosing the optimal growth model

Before analysing data regarding the impact of the COVID-19 pandemic on mental health, we evaluated four different growth models based on linear, quadratic, cubic and relative change. The evaluation showed that the growth model based on relative change demonstrated the best fit to the data for both stress levels and severity of depressive symptoms (Supplementary Table 3). We therefore used the growth model based on relative change throughout the analysis.

### Changes in stress levels

**Fig. 2:**
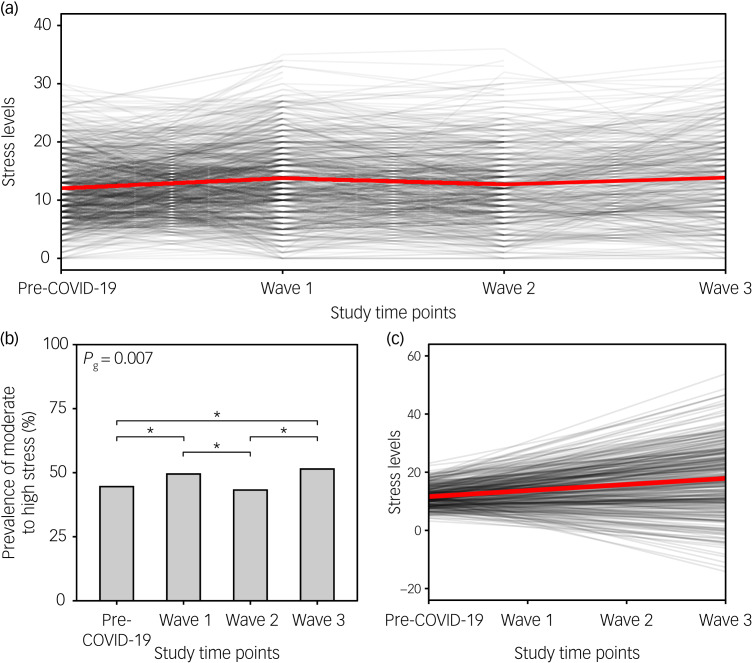
Stress levels before the COVID-19 pandemic and during the consecutive COVID-19 waves. (a) The black lines show the changes in perceived stress over the course of the COVID-19 pandemic (at individual time points) for each participant separately; the red line indicates the changes in the mean score over time points. (b) Bar plot depicts the prevalence of moderate-to-high stress (PSS score ≥14) before the pandemic and during the three COVID-19 waves. Upper horizontal bars denote significant differences between time points (*P < 0.05). (c) Spaghetti plot showing predicted individual trends of change in perceived stress during the COVID-19 pandemic for each participant (black lines), and the average trend for the whole sample (red line). PSS, Perceived Stress Scale.

We observed a significant increase in mean stress levels during the first (mean 13.8, 95% CI 13.3–14.3), second (mean 12.9, 95% CI 12.1–13.3) and third COVID-19 waves (mean 13.9, 95% CI 13.1–14.6), compared with the pre-COVID-19 period (mean PSS score 12.0, 95% CI 11.6–12.5) (Fig. 2(a)). The prevalence of moderate-to-high stress also increased from 44.6% in the pre-COVID-19 period to 49.5 and 51.4% during the first and third COVID-19 waves, respectively (Fig. 2(b)). The prevalence of moderate-to-high stress during the consecutive waves was, on average, 1.2, 1.11 and 1.22 times higher in women compared with men. The growth model (Table 2) showed that stress levels increased significantly over time (standardised *β*_slope_ = 0.473, *P* < 0.001), with respondents differing significantly in baseline stress level (standardised *β*_intercept_ = 2.681, *P* < 0.001). Both slope and intercept showed high variance (almost 50% of the total scale range), indicating large interindividual differences. The slope–intercept interaction revealed that pre-COVID-19 baseline stress levels had no direct effect on the stress level changes during the pandemic (std. covariance_(slope,intercept)_ = 0.057, *P* = 0.743) (Fig. 2(c)).

**Table 2:** Latent growth curve model goodness-of-fit indices and main estimates for severity of depressive symptoms and stress level

	Stress level				Severity of depressive symptoms			
	Value				Value			
*χ*^2^ (d.f.)	16.043 (3)				8.855 (6)			
*χ*^2^ *P*-value	0.014				0.182			
AIC	7856.392				4625.77			
BIC	7886.413				4657.056			
CFI	0.98				0.987			
TLI	0.98				0.987			
RMSEA	0.078				0.041			
	Estimate (95% CI)	Estimated variance (s.e.)	Standardised *β*	*P-*value	Estimate (95% CI)	Estimated variance (s.e.)	Standardised *β*	*P-*value
Slope (s)	2.088 (1.179–2.997)	19.510 (0.464)	0.473	<0.001	0.847 (0.693–0.999)	0.435 (0.078)	1.284	<0.001
Intercept (i)	11.611 (10.964–12.258)	18.757 (0.330)	2.681	<0.001	0.585 (0.474–0.695)	0.287 (0.056)	1.091	<0.001
Covariance (s,i)	1.083 (−5.390 to 7.557)	(3.303)	0.057	0.743	0.277 (0.055–0.499)	(0.113)	0.784	0.014

AIC, Akaike information criterion; BIC, Bayesian information criterion; CFI, Comparative Fit Index; TLI, Tucker–Lewis index; RMSEA, root-mean-square error of approximation.

### Changes in the severity of depressive symptoms

**Fig. 3:**
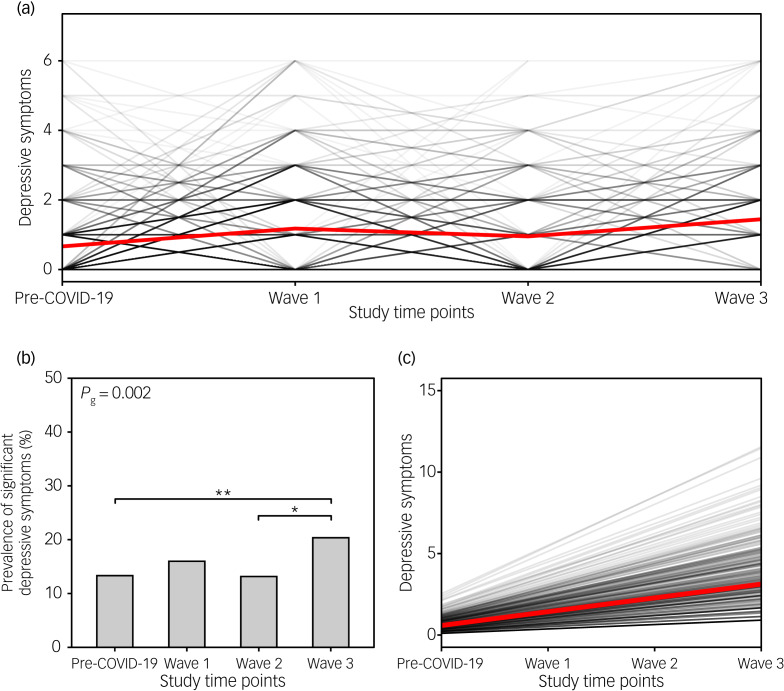
Severity of depressive symptoms before the COVID-19 pandemic and during the consecutive COVID-19 waves. (a) The black lines show the changes in the severity of depressive symptoms over the course of the COVID-19 pandemic (at individual time points) for each participant separately; the red line indicates the changes in the mean score over time points. (b) Bar plot depicts the prevalence of significant depressive symptoms (PHQ score ≥3) before the pandemic and during the three COVID-19 waves. Upper horizontal bars denote significant differences between time points (*P < 0.05, **P < 0.01). (c) Spaghetti plot showing predicted individual trends of change in severity of depressive symptoms during the COVID-19 pandemic for each participant (black lines), and the average trend for the whole sample (red line). PHQ, Patient Health Questionnaire.

Similarly, the severity of depressive symptoms increased during the first (mean 1.2, 95% CI 1.1–1.3), second (mean 1.0, 95% CI 0.8–1.1) and third COVID-19 waves (mean 1.4, 95% CI 1.3–1.6), compared with the pre-COVID-19 period (mean 0.7, 95% CI 0.6–0.7) (Fig. 3(a)). The prevalence of significant depressive symptoms increased from 13.3% in the pre-COVID-19 period to 16 and 20.4% during the first and third COVID-19 waves, respectively (Fig. 3(b)). Comparable to stress levels, the prevalence of significant depressive symptoms during the consecutive waves was, on average, 1.28, 1.11 and 1.31 times higher in women compared with men, respectively. The growth model (Table 2) confirmed these findings by showing that the severity of depressive symptoms increased significantly over time (standardised *β*_slope_ = 1.284, *P* < 0.001). Individual respondents also differed in the severity of baseline depressive symptoms (standardised *β*_intercept_ = 1.091, *P* < 0.001). Those showing more severe depressive symptoms at baseline exhibited the most significant increase in the severity of depressive symptoms over the course of the pandemic (std. covariance_(slope,intercept)_ = 0.784, *P* = 0.014) (Fig. 3(c)). Slope and intercept variance were significantly lower (7 and 5% of the scale range, respectively) compared with the values obtained for stress levels, suggesting greater interindividual similarities.

### The effects of primary and secondary stressors

**Fig. 4:**
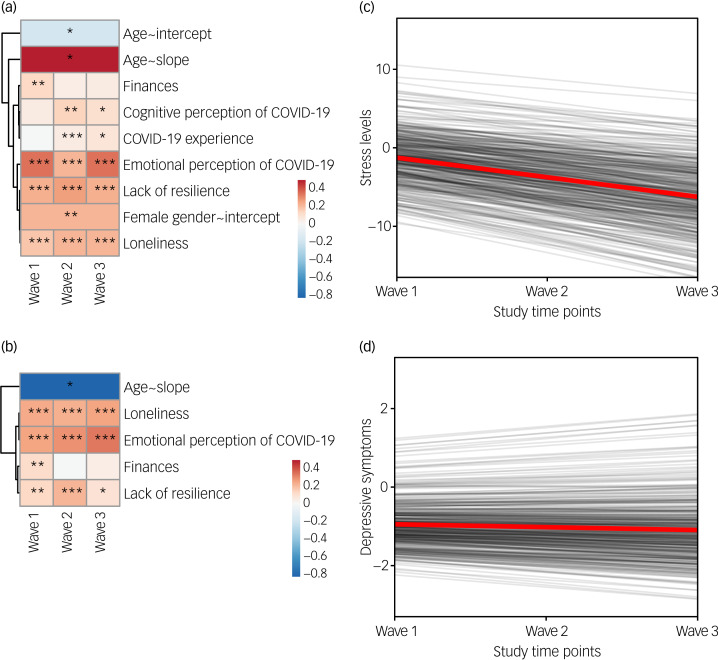
Effect of secondary stressors on longitudinal changes in stress level and severity of depressive symptoms during the consecutive COVID-19 waves. (a) Heatmap showing standardised beta coefficients of the effect of time-variant and time-invariant secondary stressors on stress levels (only stressors with a significant effect are included). Asterisks indicate the level of significance (*P < 0.05, **P < 0.01, ***P < 0.001). (b) Heatmap showing standardised beta coefficients of the effect of time-variant secondary stressors on severity of depressive symptoms (only stressors with a significant effect are included). Asterisks indicate the level of significance (*P < 0.05, **P < 0.01, ***P < 0.001). (c) Spaghetti plot showing predicted individual trends of change in perceived stress during the COVID-19 pandemic for each participant (black lines), and the average trend for the whole sample (red line) when controlling for the effect of significant secondary stressors (shown in (a)). (d) Spaghetti plot showing predicted individual trends of change in severity of depressive symptoms during the COVID-19 pandemic for each participant (black lines), and the average trend for the whole sample (red line) when controlling for the effect of significant secondary stressors (shown in (c)).

Finally, we investigated the potential effects of psychosocial variables and lifestyle factors on the development of mental health difficulties during the COVID-19 pandemic. Results showed that feelings of loneliness, emotional perception of COVID-19 as threatening and being older had the greatest effect on the increase in perceived stress. Conversely, higher levels of resilience served as a buffer against increased stress. Although men had lower baseline stress levels, the change in stress levels over time did not differ between men and women. Finally, we also observed a negative effect of a poor financial situation during the first wave and higher stress levels among the respondents who emotionally perceived COVID-19 as threatening during the second and third waves (Fig. 4(a), Table 3).

**Table 3:** Latent growth curve model goodness-of-fit and estimates of the effect of risk factors on longitudinal changes in the severity of depressive symptoms and stress level

	Stress level				Severity of depressive symptoms			
	Value				Value			
*χ*^2^ (d.f.)	96.471 (38)				50.239 (28)			
*χ*^2^ *P*-value	<0.001				0.006			
AIC	6549.68				3356.535			
BIC	6659.182				3446.483			
CFI	0.937				0.950			
TLI	0.901				0.920			
RMSEA	0.066				0.049			
	Estimate (95% CI)	Estimated variance (s.e.)	Standardised *β*	*P-*value	Estimate (95% CI)	Estimated variance (s.e.)	Standardised *β*	*P-*value
Slope (s)	−5.131 (−8.880 to −1.382)	1.750 (1.913)	−3.409	0.007	−0.201 (−0.835 to 0.432)	0.002 (0.364)	2.064	0.639
Intercept (i)	2.939 (−0.574 to 6.451)	12.146 (1.792)	0.815	0.101	0.592 (−0.088 to 1.273)	0.396 (0.301)	−0.978	0.037
Covariance (s,i)	0.418 (−5.170 to 6.005)	(2.851)	0.091	0.883	0.075 (−0.083 to 0.234)	(0.106)		
Time invariant risk factors								
Age~slope	0.055 (0.007, 0.104)	0.025	0.482	0.025	−0.005 (−0.016 to 0.006)	0.006	−0.827	0.026
Gender (female)~slope	−0.13 (−1.744, 1.484)	0.824	−0.43	0.875	0.02 (−0.119 to 0.158)	0.071	0.118	0.179
Age~intercept	−0.052 (−0.103, −0.001)	0.026	−0.19	0.045	−0.007 (−0.018 to 0.003)	0.005	−0.147	0.781
Gender (female)~intercept	1.508 (0.355, 2.662)	0.583	0.207	0.01	0.189 (0.023–0.354)	0.085	0.146	0.352
Time-variant risk factors								
Finances (wave 1)	1.087 (0.468–1.706)	0.316	0.123	0.001	0.205 (0.05–0.36)	0.079	0.115	0.009
Loneliness (wave 1)	0.664 (0.369–0.96)	0.151	0.169	<0.001	0.182 (0.116–0.249)	0.034	0.230	<0.001
Resilience (wave 1)	1.178 (0.808–1.548)	0.189	0.226	<0.001	0.138 (0.04–0.236)	0.050	0.131	0.006
Emotional perception of COVID-19 (wave 1)	0.513 (0.375–0.652)	0.071	0.344	<0.001	0.074 (0.049–0.1)	0.013	0.246	<0.001
Cognitive perception of COVID-19 (wave 1)	0.058 (−0.011 to 0.126)	0.035	0.054	0.098				
Finances (wave 2)	0.339 (−0.278 to 0.955)	0.315	0.037	0.282	−0.003 (−0.194 to 0.188)	0.098	−0.002	0.977
Loneliness (wave 2)	0.92 (0.596–1.243)	0.165	0.214	<0.001	0.182 (0.084–0.28)	0.050	0.218	<0.001
Resilience (wave 2)	1.3 (0.857–1.744)	0.226	0.254	<0.001	0.214 (0.111–0.316)	0.052	0.215	<0.001
Emotional perception of COVID-19 (wave 2)	0.325 (0.196–0.454)	0.066	0.213	<0.001	0.083 (0.055–0.111)	0.014	0.280	<0.001
Cognitive perception of COVID-19 (wave 2)	0.153 (0.065–0.241)	0.045	0.139	0.001				
COVID-19 experience (wave 2)	4.514 (3.052–5.976)	0.746	0.052	<0.001				
Finances (wave 3)	0.57 (−0.181 to 1.32)	0.383	0.059	0.137	0.094 (−0.106 to 0.294)	0.102	0.046	0.356
Loneliness (wave 3)	0.832 (0.542–1.123)	0.148	0.214	<0.001	0.203 (0.125–0.281)	0.040	0.250	<0.001
Resilience (wave 3)	1.343 (0.916–1.77)	0.218	0.223	<0.001	0.12 (0.008–0.233)	0.057	0.096	0.035
Emotional perception of COVID-19 (wave 3)	0.518 (0.384–0.651)	0.668	0.342	<0.001	0.103 (0.07–0.136)	0.017	0.325	<0.001
Cognitive perception of COVID-19 (wave 3)	0.103 (0.004–0.201)	0.050	0.097	0.041				
COVID-19 experience (wave 3)	1.429 (0.221–2.638)	0.617	0.082	0.02				

AIC, Akaike information criterion; BIC, Bayesian information criterion; CFI, Comparative Fit Index; TLI, Tucker–Lewis index; RMSEA, root-mean-square error of approximation.

The severity of depressive symptoms was affected by fewer secondary stressors. We again observed a negative effect of feelings of loneliness and negative emotional perception of the COVID-19 illness throughout the pandemic, and more severe depressive symptoms with worse financial situation during the first wave. Furthermore, we found a protective effect of the higher resilience against increased severity in depressive symptoms. Finally, older respondents demonstrated a greater decline in the severity of depressive symptoms over time than younger respondents (Fig. 4(b), Table 3).

Intriguingly, when we controlled for the effects of secondary stressors, increased stress levels during the COVID-19 pandemic were no longer detectable. We rather observed a significant decrease in stress levels, with lower variance (standardised *β* = –3.409, *P* = 0.007, *σ*^2^ = 1.750) (Fig. 4(c)). Similarly, we noticed that the severity of depressive symptoms did not change over time in this context, and remained essentially identical for all the respondents during the pandemic (standardised *β* = 2.064, *P* = 0.639, *σ*^2^ = 0.002) (Fig. 4(d)).

## Discussion

The aim of this study was to investigate the long-term impact of the COVID-19 waves on mental health. Given that understanding the mechanisms underlying possible changes in mental health during the pandemic is essential for the formulation of adequate interventions, we also examined the effects of potential stressors on mental health during the COVID-19 pandemic. To achieve this objective, we first computed the prevalence of stress levels and the severity of depressive symptoms, and then employed an LGCM to evaluate changes in the trajectories of stress levels and severity of depressive symptoms during the pandemic. This approach had several advantages. First, compared with analysis-of-variance models, the LGCM allows for analysing changes in the observed parameters not only at the general (average) level, but also at the individual level. Second, adding the covariates (secondary stressors) to the LGCM reveals the impact of these covariates and indicates the degree of direct impact of the COVID-19 pandemic (as primary stressor) over time on mental health difficulties.

Our findings showed that stress levels and severity of depressive symptoms increased significantly during the COVID-19 pandemic, and the prevalence of increased stress and significant depressive symptoms was greater during the pandemic compared with pre-pandemic levels, with a greater increase in women than men. Self-reported repeated testing of parameters indicative of mental health difficulties before and across the COVID-19 waves in the Czech Republic provided results consistent with several similar longitudinal studies reported recently from other countries, including the UK,^6,8^ Germany,^42^ Canada,^7^ Switzerland^43^ and France,^3^ as well as a recent meta-analytical study^44^ and numerous cross-sectional studies.^45,46^ The gender-related differences were also consistent with previous studies.^47,48^ The possible reasons reported for these differences are the stronger emotional response in women and the greater impact of pandemic measures on women's everyday life and in maintaining a balance between family and work (especially when caring for children).^49^ The LGCM provided an extended perspective, confirming a significant increase in mental health difficulties during the pandemic when only the development of these primary outcomes was examined. However, it demonstrated relatively high variability in both slopes and intercepts, suggesting significant interindividual differences among respondents. These differences in individual trajectories suggest that the ability to cope with the burden imposed by the COVID-19 pandemic is, to some extent, dependent on the influence of other stressors and on the overall level of coping resources available to the individual, and their durability or rate of depletion in coping with the pandemic.^5^

Intriguingly, when we included the effects of primary and secondary stressors in the growth models, a very different pattern emerged. We identified several stressors that influenced the level of stress and severity of depressive symptoms. The stressors that negatively affected both indicators of mental health difficulties were associated with the emotional experience of the circumstances of the COVID-19 pandemic; namely, feelings of loneliness and the negative effect of COVID-19 disease on emotions. These results support previous findings of a significant link between negative emotionality and an increase in mental health difficulties.^6,9,10,12,50^ Resilience, on the other hand, demonstrated a protective, buffering effect, consistent with its very definition, which is the ability to overcome adversity without compromising one's own mental and emotional stability.^45,51,52^

Besides these common negative factors, we identified several other specific acting stressors. In older respondents, we observed a lower drop in stress levels over time (although still with a decreasing tendency), but at the same time, a more pronounced decrease in the severity of depressive symptoms compared with the younger population. The worse outcomes in the younger population are likely to be associated with disrupted social interactions, greater worries about studies, job security and financial stability, and enriched life experiences and reduced life expectations in older participants.^44,53^ Finally, in the first wave of the COVID-19 pandemic, the deterioration of the financial situation also had a negative impact. As this negative effect was no longer evident in subsequent waves, the adaptation to the new conditions based on experience, together with the support measures put in place by the governments (such as the furlough scheme), seems to have eliminated this stressor.

Compared with the initial simple growth models (with only primary outcomes), when including primary and secondary stressors, the longitudinal effect of the COVID-19 pandemic on mental health difficulties completely disappears. The severity of depressive symptoms has now remained constant over time, whereas for stress levels we can even observe a significant decrease over the course of the COVID-19 pandemic. In all cases (i.e. for both indicators of mental health difficulties separately and taking into account stressors), pre-pandemic levels of stress and depressive symptoms proved to be a significant predictor of their peri-pandemic development. This suggests that the baseline level of resources available to cope with challenging situations (or level of exhaustion) plays a pivotal role,^5^ and the ability to identify at-risk individuals based on pre-existing difficulties may be important in minimising the impact of a pandemic on mental health. In addition, the variability of slopes for both outcomes also decreased significantly. These results suggest that the observed increase in mental health difficulties during the COVID-19 pandemic is largely attributable to the effect of secondary stressors associated with, and resulting from, the pandemic, and not only to the long-term occurrence of the pandemic itself (given the primary stressors showed only a partial impact).

This finding has significant implications for the interventions designed to safeguard mental health during pandemics. Although we may not be able to eliminate pandemics swiftly, the secondary stressors responsible for mental health difficulties are present before the onset of pandemics and are readily amenable to interventions. This makes it possible to focus support and care on eliminating the negative impact of these stressors, thereby reducing the rate of mental health difficulties in a population before to a pandemic.

### Strengths and limitations

Our study has several strengths. First, we describe changes in mental health difficulties in several distinct waves of the COVID-19 pandemic that differ in their characteristics, severity, related measures, duration and impact on people's daily lives. In addition, we compare these changes with the situation before the start of the COVID-19 pandemic. Second, using growth models, we describe long-term changes in mental health difficulties not only in terms of average values, but also at the individual level of each respondent, including how initial levels of mental health difficulties are reflected in their evolution during the COVID-19 pandemic. Third, and most importantly, in contrast to most other large population surveys and research studies, we analyse the impact of multiple key secondary stressors at once, measured using standardised psychological instruments. Compared with many other studies that have included only the influence of basic sociodemographic characteristics or one or two risk factors, we can portray a more comprehensive picture of the longitudinal evolution of mental health during a pandemic, and therefore outline areas that require further research in larger or socially and culturally diverse populations.

There are also several limitations that should be mentioned. Although the observations about mental health before and during the three consecutive COVID-19 waves reported in this study derive from a probability population-based sample, the examined sample size of 788 participants is relatively small compared with national surveys^4^ and some other studies.^6,42^ Although this is a representative population sample, the smaller number of respondents may affect the generalisability of the described findings, which would benefit from further confirmation on larger samples. The drop-out rates recorded in our study, however, are lower than the ones reported in other longitudinal studies of mental health before and/or during the COVID-19 waves.^6,42,54^ Similar to other studies,^6,54^ we also used self-reported psychological scales and questionnaires to estimate changes in mental health in response to consecutive COVID-19 waves. Therefore, our results are based on the examination of symptoms rather than formal clinical diagnoses of mental health disorders. As our COVID-19 add-on cohort was sampled exclusively during the COVID-19 waves, our study precludes testing whether mental distress observed during the COVID-19 waves subsided in the time intervals between the COVID-19 waves. Finally, given the small cohort size, further studies are needed to confirm the significant or perhaps exclusive role of the secondary stressors on mental health during the pandemic.

To summarise, we show that increased stress levels and severity of depressive symptoms observed at the onset of the COVID-19 pandemic compared with the pre-COVID-19 period persist throughout all consecutive COVID-19 waves. This finding is indicative of the long-term effects of the COVID-19 pandemic on mental health. Our results suggest that mental health difficulties during the COVID-19 pandemic are attributable mainly to the effect of secondary stressors. This suggests that not only do these stressors play a pivotal role in the experience of a pandemic, but that their continued presence may also have a major impact on how quickly mental health difficulties recede after a pandemic ends. Moreover, given that pre-pandemic levels of stress and depressive symptoms were significant predictors of future mental health outcomes, the ability to identify at-risk individuals based on pre-existing difficulties may be an important tool to minimise the impact of future pandemics. Taken together, the incorporation of more efficient and targeted approaches to mental healthcare, more timely strategies to identify and treat individuals at high risk for developing mental health difficulties,^55,56^ and a proactive use of online technologies and web-based intervention tools^57^ could help curb mental health difficulties that have arisen during the COVID-19 pandemic.

## Supporting information

Novotný et al. supplementary material Novotný et al. supplementary material

## Data Availability

The data that support the findings of this study are available from the corresponding author, G.B.S., upon substantiated request and approval by the St. Anne's University Hospital International Clinical Research Centre internal board.
