# Supplementary material for: The long-term effects of consecutive COVID-19 waves on mental health
Source: BJPsych Open. 2023 Dec 19;10(1):e15. doi: 10.1192/bjo.2023.620 (PMC10755548; doi:10.1192/bjo.2023.620)
Supplement: Novotný et al. supplementary material [file S2056472423006208sup001.pdf]

Supplementary Appendix for

'The adverse impact of consecutive COVID-19 waves on mental health'

Supplementary Table 1. Selected supporting evidence for inclusion of individual risk factors

Risk factor	References
**COVID-19 perception**	• Costa EC V., Vale S, Sobral M, Graça Pereira M. Illness perceptions are the main predictors of depression and anxiety symptoms in patients with chronic pain. *Psychol Health Med*. 2016;21(4):483-495. doi:10.1080/13548506.2015.1109673<br>• Belen H. Fear of COVID-19 and Mental Health: The Role of Mindfulness in During Times of Crisis. *Int J Ment Health Addict*. 2022;20(1):607-618. doi:10.1007/s11469-020-00470-2<br>• Rodríguez-Hidalgo AJ, Pantaleón Y, Dios I, Falla D. Fear of COVID-19, Stress, and Anxiety in University Undergraduate Students: A Predictive Model for Depression. *Front Psychol*. 2020;11. doi:10.3389/fpsyg.2020.591797
**Exposure to COVID-19**	• Mak IWC, Chu CM, Pan PC, Yiu MGC, Chan VL. Long-term psychiatric morbidities among SARS survivors. *Gen Hosp Psychiatry*. 2009;31(4):318-326. doi:10.1016/j.genhosppsych.2009.03.001<br>• Hawryluck L, Gold WL, Robinson S, Pogorski S, Galea S, Styra R. SARS Control and Psychological Effects of Quarantine, Toronto, Canada. *Emerg Infect Dis*. 2004;10(7):1206-1212. doi:10.3201/eid1007.030703
**Loneliness**	• Killgore WDS, Cloonan SA, Taylor EC, Dailey NS. Loneliness: A signature mental health concern in the era of COVID-19. *Psychiatry Res*. 2020;290:113117. doi:10.1016/j.psychres.2020.113117<br>• Wang J, Mann F, Lloyd-Evans B, Ma R, Johnson S. Associations between loneliness and perceived social support and outcomes of mental health problems: A systematic review. *BMC Psychiatry*. 2018;18(1). doi:10.1186/s12888-018-1736-5<br>• Rosenberg M, Luetke M, Hensel D, Kianersi S, Fu T chieh, Herbenick D. Depression and loneliness during April 2020 COVID-19 restrictions in the United States, and their associations with frequency of social and sexual connections. *Soc Psychiatry Psychiatr Epidemiol*. 2021;56(7):1221-1232. doi:10.1007/s00127-020-02002-8<br>• Cacioppo JT, Hughes ME, Waite LJ, Hawkley LC, Thisted RA. Loneliness as a specific risk factor for depressive symptoms: Cross-sectional and longitudinal analyses. *Psychol Aging*. 2006;21(1):140-151. doi:10.1037/0882-7974.21.1.140<br>• Fernández RS, Crivelli L, Guimet NM, Allegri RF, Pedreira ME. Psychological Distress associated with COVID-19 quarantine: Latent Profile Analysis, Outcome Prediction and Mediation Analysis. *J Affect Disord*. 2020;277:75. doi:10.1016/j.jad.2020.07.133<br>• Bonanno GA, Ho SMY, Chan JCK, et al. Psychological resilience and dysfunction among hospitalized survivors of the SARS epidemic in Hong Kong: A latent class approach. *Heal Psychol*. 2008;27(5):659-667. doi:10.1037/0278-6133.27.5.659
**Resilience**	• Olff M, Primasari I, Qing Y, et al. Mental health responses to COVID-19 around the world. *Eur J Psychotraumatol*. 2021;12(1):1929754. doi:10.1080/20008198.2021.1929754<br>• Petzold MB, Bendau A, Plag J, et al. Risk, resilience, psychological distress, and anxiety at the beginning of the COVID-19 pandemic in Germany. Brain Behav. Published online July 7, 2020. doi:10.1002/brb3.1745<br>• Killgore WDS, Taylor EC, Cloonan SA, Dailey NS. Psychological resilience during the COVID-19 lockdown. *Psychiatry Res*. 2020;291:113216. doi:10.1016/j.psychres.2020.113216<br>• Bonanno GA, Ho SMY, Chan JCK, et al. Psychological resilience and dysfunction among hospitalized survivors of the SARS epidemic in Hong Kong: A latent class approach. *Heal Psychol*. 2008;27(5):659-667. doi:10.1037/0278-6133.27.5.659
**Finances**	• Wilson JM, Lee J, Fitzgerald HN, Oosterhoff B, Sevi B, Shook NJ. Job Insecurity and Financial Concern During the COVID-19 Pandemic Are Associated With Worse Mental Health. *J Occup Environ Med*. 2020;62(9):686-691. doi:10.1097/JOM.0000000000001962<br>• Witteveen D, Velthorst E. Economic hardship and mental health complaints during COVID-19. *Proc Natl Acad Sci*. 2020;117(44):27277-27284. doi:10.1073/pnas.2009609117
**Physical activity**	• Ai X, Yang J, Lin Z, Wan X. Mental Health and the Role of Physical Activity During the COVID-19 Pandemic. *Front Psychol*. 2021;12. doi:10.3389/fpsyg.2021.759987<br>• Grocke-Dewey M, Hardison-Moody A, Haynes-Maslow L, et al. Examining the relationship between physical activity and mental health during the COVID-19 pandemic across five U.S. States. *Prev Med Reports*. 2021;24:101537. doi:10.1016/j.pmedr.2021.101537
**Sleep**	• Marelli S, Castelnuovo A, Somma A, et al. Impact of COVID-19 lockdown on sleep quality in university students and administration staff. *J Neurol*. 2020;1:1. doi:10.1007/s00415-020-10056-6<br>• Pérez-Carbonell L, Meurling IJ, Wassermann D, et al. Impact of the novel coronavirus (COVID-19) pandemic on sleep. *J Thorac Dis*. 2020;12(S2):S163-S175. doi:10.21037/jtd-cus-2020-015<br>• Alimoradi Z, Broström A, Tsang HWH, et al. Sleep problems during COVID-19 pandemic and its' association to psychological distress: A systematic review and meta-analysis. *EClinicalMedicine*. 2021;36:100916. doi:10.1016/j.eclinm.2021.100916<br>• Partinen M, Holzinger B, Morin CM, et al. Sleep and daytime problems during the COVID-19 pandemic and effects of coronavirus infection, confinement and financial suffering: a multinational survey using a harmonised questionnaire. *BMJ Open*. 2021;11(12):e050672. doi:10.1136/bmjopen-2021-050672
**Age**	• Russo C, Terraneo M. Mental Well-being Among Workers: A Cross-national Analysis of Job Insecurity Impact on the Workforce. *Soc Indic Res*. Published online July 21, 2020. doi:10.1007/s11205-020-02441-5<br>• Patel K, Robertson E, Kwong ASF, et al. Psychological Distress Before and During the COVID-19 Pandemic Among Adults in the United Kingdom Based on Coordinated Analyses of 11 Longitudinal Studies. *JAMA Netw Open*. 2022;5(4):e227629. doi:10.1001/jamanetworkopen.2022.7629

	• Liu CH, Zhang E, Wong GTF, Hyun S, Hahm H "Chris." Factors associated with depression, anxiety, and PTSD symptomatology during the COVID-19 pandemic: Clinical implications for U.S. young adult mental health. *Psychiatry Res*. 2020;290:113172. doi:10.1016/j.psychres.2020.113172<br>• Jia R, Ayling K, Chalder T, et al. The prevalence, incidence, prognosis and risk factors for symptoms of depression and anxiety in a UK cohort during the COVID-19 pandemic. *BJPsych Open*. 2022;8(2):e64. doi:10.1192/bjo.2022.34
**Sex**	• Killgore WDS, Cloonan SA, Taylor EC, Dailey NS. Loneliness: A signature mental health concern in the era of COVID-19. *Psychiatry Res*. 2020;290:113117. doi:10.1016/j.psychres.2020.113117<br>• Zamarro G, Prados MJ. Gender differences in couples' division of childcare, work and mental health during COVID-19. *Rev Econ Househ*. 2021;19(1):11-40. doi:10.1007/s11150-020-09534-7<br>• Ramiz L, Contrand B, Rojas Castro MY, et al. A longitudinal study of mental health before and during COVID-19 lockdown in the French population. *Global Health*. 2021;17(1):29. doi:10.1186/s12992-021-00682-8

Supplementary Table 2. Comparison of the goodness-of-fit of full base model with final model

	**A. Stress level**			**B. Severity of depressive symptoms**		
	**Base model**	**Final model**	**Comparison**	**Base model**	**Final model**	**Comparison**
X^2 [DF]	134.528[61]	96.471[38]	40.34[23]	112.709[61]	50.239[28]	57.145[33]
X^2 P-value	<0.001	<0.001	0.014	<0.001	0.006	0.006
AIC	7591.865	6549.68		4433.194	3356.535	
BIC	7715.7	6659.182		4562.25	3446.483	
CFI	0.92	0.937		0.907	0.95	
TLI	0.887	0.901		0.869	0.92	
RMSEA	0.062	0.066		0.048	0.049	

Supplementary Table 3. Comparison of the goodness-of-fit of individual growth model formulas

	**AIC**	**BIC**	**CFI**	**TLI**	**RMSEA**
**Depressive symptoms**					
Linear	4670.62	4694	0.83	0.873	0.129
Quadratic	4682.75	4706.22	0.79	0.842	0.144
Cubic	4686.75	4710.21	0.776	0.832	0.148
Relative change	4625.77	4657.06	0.987	0.987	0.041
**Stress level**					
Linear	7899.72	7922.23	0.903	0.927	0.148
Quadratic	7918.33	7940.84	0.87	0.902	0.171
Cubic	7922.36	7944.87	0.863	0.897	0.176
Relative change	7856.39	7886.41	0.98	0.98	0.078

Kardiovize COVID-19 e-questionnaire (excluding standardized questionnaires)

If the question was used only in one of the data collections or has been modified, this is indicated in italics in square brackets after the question text)

1) What is your current weight? *[Data collection 1]*
2) How many cigarettes do you smoke per day? If you are a non-smoker, please, fill 0. *[Data collection 1]*
3) What is your current family situation?
   a. Living in the relationship with the children
   b. Living in the relationship without the children
   c. Monoparental household (living with one parent)
   d. Living alone
   e. Other (please, specify)
4) How many children do you have? *[Data collection 1]*
   a. None
   b. One child
   c. Two children
   d. Three or more children
5) Who are you spending your time with during the quarantine? (multiple choice)
   a. No one
   b. With my partner or spouse
   c. With my children
   d. With other family members
   e. With someone outside my own family
6) During the last 14 days, how often have you been actively and specifically seeking information about the current situation regarding the COVID-19 pandemic and related measures?
   a. Never
   b. Less than once per week
   c. 1-2 times per week
   d. 2-3 times per week
   e. Approximately once per day
   f. Many times per day
7) Does the COVID-19 state of emergency affect your financial situation?
   a. Not at all
   b. Just a little
   c. Pretty much
   d. Extremely
8) How much does the current COVID-19 situation affect your work life? (multiple choice)
   a. The pandemic did not affect my work life/I am currently not working
   b. I have more work than usual
   c. I have less work than usual
   d. I work from home
   e. I changed my job/my job duties or position changed
   f. I stayed home because of kids or family member
   g. I lost my job
9) How many individual PRIVATE (not work-related) social contacts (phone, SMS, Skype, WhatsApp, email, ...) have you had in the last 7 days? *[Data collection 1]*
   a. None, I am without social contacts
   b. 1 to 3 contacts
   c. 4 -7 contacts
   d. 8 -14 contacts
   e. 15 and more contacts
10) How many individual WORK-RELATED social contacts (phone, SMS, Skype, WhatsApp, email, ...) have you had in the last 7 days? *[Data collection 1]*
   a. None
   b. 1 to 3 contacts
   c. 4 -7 contacts
   d. 8 -14 contacts
   e. 15 and more contacts

11) Has your sleep quality changed in the past last 14 days/compared to last time you filled this survey?
    a. it got better
    b. it not changed
    c. it got worse
12) Has the length of your sleep changed (on average per day) /compared to last time you filled this survey?
    a. sleep time has increased
    b. sleep time did not change
    c. sleep time has decreased

How often have you exercised in the last 14 days? Write down how many hours per week have you spent performing specific exercises, if zero time, fill in 0.

13. Low intensity exercise (e.g. walking):
14. High intensity exercise (e.g. running):
15. Body building:
16. Stretching:
17. Has the frequency of how often you exercise changed over the last 14 days/compared to last time you filled this survey?
    a. the frequency has increased
    b. the frequency has not changed
    c. the frequency has decreased
18. How many times per week did you go out from your home (work, shop, nature, etc.) in the last 14 days?
    a. Never
    b. 1-2 times per week
    c. 3-5 times per week
    d. Almost every day

How do you follow the government-imposed COVID-19 state of emergency measures?

19. Are you wearing a mask/respirator?
    a. Always
    b. Almost always
    c. Sometimes
    d. Never
20. How often are you washing or disinfecting your hands?
    a. Always
    b. Almost always
    c. Sometimes
    d. Never
21. How often have you respected the restriction of going out?
    a. Always
    b. Almost always
    c. Sometimes
    d. Never
22. How often have you respected the 2-meter social distancing?
    a. Always
    b. Almost always
    c. Sometimes
    d. Never
23. How often have you respected the ban of direct contact with other people?
    a. Always
    b. Almost always
    c. Sometimes
    d. Never
24. How often have you respected the measure that only two people can be in closer contact in public places?
    a. Always
    b. Almost always
    c. Sometimes
    d. Never
25. Do you trust the government and other institutions involved in how they are dealing with the current situation? [*Data collections 2 and 3]*

1 = Do not trust at all - 5 = Absolutely trust

26. Are current anti-epidemic measures adequate? [*Data collections 2 and 3]*

1 = Completely inadequate - 5 = Completely adequate

27. When do you think the life will get back to the normal in the Czech Republic*? Please indicate the number of months.*

28. How many days did you spend in isolation?
If you have not been in quarantine, please fill in 0.
    a. because of contact with a person with confirmed coronavirus infection:
    b. because returning from a COVID-19 high risk country:
    c. because you have been tested positive for coronavirus infection:
29. Have you been vaccinated with the Covid-19 vaccine? *[Data collection 3]*
    a. Yes
    b. No
30. What is your willingness to be vaccinated with the Covid-19 vaccine? *[Data collection 3]*
    1 = Refuse to be vaccinated - 5 = Definitely want to be vaccinated
31. Have you fell ill with COVID-19?
    a. Yes
    b. No
32. What symptoms or signs of COVID-19 have you manifested? (multiple choice)
    c. Fever
    d. Runny nose and cough
    e. Emphysema
    f. Pneumonia
    g. Loss of taste and smell
    h. Headache and dizziness
    i. Nausea, vomiting, diarrhea
    j. Weakness, joints and muscle pain
    k. No symptoms or signs

In the following section we will ask you about your health. *[Data collection 1]*

33. Have you been treated for arterial hypertension?
    a. Yes
    b. No
34. If yes, please provide the name of the medication and the dosage to treat arterial hypertension:
35. Have you been diagnosed with diabetes mellitus type I?
    a. Yes
    b. No
36. If yes, please provide the name of the medication and the dosage to treat diabetes mellitus type I:
37. Have you been diagnosed for diabetes mellitus type II?
    a. Yes
    b. No
38. If yes, please provide the name of the medication and the dosage to treat diabetes mellitus type II:
39. Have you been diagnosed with disease of respiratory-tract (astma bronchiale, CHOPN, etc.)?
    a. Yes
    b. No
40. If yes, please provide the name of the medication and the dosage to treat the respiratory-track disease:
41. Have you been diagnosed with any of the following immune disorders?
    a. Inflammatory bowel disease (e.g. ulcerative colitis or Crohn's disease)
    b. Rheumatic diseases (e.g. rheumatoid arthritis)
    c. Multiple sclerosis (MS)
    d. Bone marrow transplant
    e. Organ transplant and immunosuppressive therapy
    f. Cancer treated with chemotherapy or radiotherapy
    g. None
42. If yes, please provide the name of the medication and the dosage to treat these disorders:
43. Have you been diagnosed with an allergy or atopic eczema?
    a. Yes
    b. No

44. Are you currently taking medicines containing corticosteroid (e.g. Decamed, Medrol, Depo-Medrol, Dexamethasone, Hydrocortisone, Fortecortin, Methycetone, Fludrocortisone)?
    a. Yes
    b. No
45. Are you currently taking medicines containing Hydrochloroquine (e.g. Plaquenil)?
    a. Yes
    b. No
